# Medical oncology future plan of the Spanish Society of Medical Oncology: challenges and future needs of the Spanish oncologists

**DOI:** 10.1007/s12094-016-1595-9

**Published:** 2016-12-22

**Authors:** F. Rivera, R. Andres, E. Felip, R. Garcia-Campelo, P. Lianes, A. Llombart, J. M. Piera, J. Puente, C. A. Rodriguez, R. Vera, J. A. Virizuela, M. Martin, P. Garrido

**Affiliations:** 1 Medical Oncology Department, Hospital Universitario Marqués de Valdecilla, Santander, Spain; 2 Medical Oncology Department, Hospital Clínico Lozano Blesa, Zaragoza, Spain; 3 Institut d’Oncologia, Vall d’Hebron University Hospital, Barcelona, Spain; 4 Medical Oncology Department, Complejo Hospitalario Universitario A Coruña, Coruña, Spain; 5 Medical Oncology Department, Hospital de Mataró, Mataró, Barcelona Spain; 6 Medical Oncology Department, Hospital Universitàri Arnau de Vilanova, Lleida, Spain; 7 Medical Oncology Department, University Hospital Donostia, Donostia/San Sebastián, Spain; 8 Medical Oncology Department, Hospital Clínico Universitario San Carlos, Madrid, Spain; 9 Medical Oncology Department, Hospital Clínico Universitario, Salamanca, Spain; 10 Medical Oncology Department, Complejo Hospitalario de Navarra, Pamplona, Spain; 11 Medical Oncology Department, Complejo Hospitalario Regional Virgen Macarena, Sevilla, Spain; 12 Department of Medical Oncology, Instituto de Investigación Sanitaria Gregorio Marañón, Hospital General Universitario Gregorio Marañón, Madrid, Spain; 13 Department of Medical Oncology, IRYCIS, Hospital Universitario Ramón y Cajal, Madrid, Spain; 14 Sociedad Española de Oncología Médica (SEOM), C/ Velázquez, 7-3º planta, 28001 Madrid, Spain; 15 2013-2015 SEOM Executive Board, C/ Velázquez, 7-3º planta, 28001 Madrid, Spain; 16 SEOM Future Plan Advisory Board, C/ Velázquez, 7-3º planta, 28001 Madrid, Spain; 17 2015-2017 SEOM Executive Board, C/ Velázquez, 7-3º planta, 28001 Madrid, Spain

**Keywords:** Medical Oncology, Future, Spain, Workforce, Recommendations, SEOM

## Abstract

**Purpose:**

The SEOM Future Plan is aimed at identifying the main challenges, trends and needs of the medical oncology speciality over the next years, including potential oncologist workforce shortages, and proposing recommendations to overcome them.

**Methods:**

The estimations of the required medical oncologists workforce are based on an updated Medical Oncologist Register in Spain, Medical Oncology Departments activity data, dedication times and projected cancer incidence. Challenges, needs and future recommendations were drawn from an opinion survey and an advisory board.

**Results:**

A shortage of 211 FTE medical oncologist specialists has been established. To maintain an optimal ratio of 158 new cases/FTE, medical oncology workforce should reach 1881 FTE by 2035.

**Conclusions:**

Main recommendations to face the growing demand and complexity of oncology services include a yearly growth of 2.5% of medical oncologist’s workforce until 2035, and development and application of more accurate quality indicators for cancer care and health outcomes measure.

## Introduction

Advances in Medical Oncology, including earlier and more accurate diagnosis, and the discovery of more effective therapies, are contributing to improve survival rates in cancer patients and increase the number of long-term survivors [1]. Nevertheless, cancer is still one of the main concerns in Spain, as it is the leading cause of death in men and the second leading cause of death in women [2] as well as the primary cause of potential years of life lost (PYLL) [3]. In addition, the incidence of cancer in Spain continues to increase [1], due in part, to the ageing of the population, environmental factors and certain life styles in society today. The Globocan 2012 study predicts an increase of 39% in the incidence of cancer in Spain within the next 20 years, from around 227,000 new cases per year in 2015, to more than 315,000 in 2035 [4].

The increase of the incidence, the decrease of mortality rates, and the ageing of population, are causing an increase in the number of potential cancer patients who will demand treatment services at Medical Oncology Departments. This increase in demand will be accompanied by a more complex management, due both to the increase in the number of complex patients (polymedicated patients, with multiple pathologies or patients receiving several lines of chemotherapy as the main factors), and to the continuous increase of the number of drugs and indications approved for the treatment of cancer. There are currently more than 2000 drugs in development in the area of oncology, most aimed at the treatment of tumours in advanced stage or metastasis [5]. During the period of 2014 to May 2015 along, the Spanish Agency of Medicines and Medical Devices (AEMPS) approved 16 new indications in the area of cancer (either new drugs or extension of indication of already approved drugs) [6].

On the other hand, the significant progress in the field of human genomics has allowed the rapid advance in the understanding of tumour biology. Many new drugs act on molecular targets or signalling paths that correspond to specific genome alterations, which achieves more focused and effective anti-tumour activity. This knowledge is allowing the advance in the application of a “biomarker conducted” oncologic medicine or “precision medicine”. [7] The number of the so-called “accompanying diagnostics” [genetic analysis, detection of protein or metabolite levels, etc., according to the Food and Drug Administration (FDA)] is increasing. Although most of these tests are recommended by the FDA and the European Medicines Agency (EMA), clearer evidence on their clinical usefulness must still be obtained to facilitate their implantation [8].

Within this context of changes, the Spanish Society of Medical Oncology (SEOM) has considered the need of a study compiling the main future challenges of the speciality and a proposal of recommendations to respond to them with the maximum guarantee of quality in healthcare delivery and equity of access based on the best available evidence.

To adequately respond to the challenges to the speciality, it is essential to monitor potential medical oncology workforce shortages. Different studies conducted by the Ministry of Health, Social Services and Equality have periodically registered the number of professionals and have reported estimates of future needs [9]. The enactment of Royal Decree 640/2014, of the 25th of July, regulating the creation of the National Register of Health Professionals, started an important initiative that will be useful for planning the needs of qualified health professionals, as well as for coordinating public health Human Resources policies. However, in view of the lack of updated data on medical oncology specialists in Spain included in the available european study [10], and in expectation of knowing the results of the Register of Health Professionals, the Project Medical Oncology Future Plan, has produced an updated Register of Medical Oncologists (MO), which will serve as the basis for the estimation of future needs.

In addition to the workforce study, the objective of the study was to identify medical oncologists perception about the changes that are having impact in their daily practice and the main challenges and future needs of the speciality. With that purpose, an online survey was used as the basis by an Advisory Board for the issuing of recommendations on the most effective actions to be carried out.

## Materials and methods

### Register of Medical Oncologists

A register of Medical Oncology Departments, including the number of medical oncologists in public and private hospitals in Spain in 2014 was prepared. Requests for information were sent to Directorates General for Human Resources of the Regions’ Health Departments, Human Resources Departments of the main private hospitals and Hospital Groups or their equivalents. Information was collected through an electronic survey. In private hospitals, information about professionals working simultaneously in private and public sectors was collected to avoid duplication of data.

Information was collected from 202 public and private centres. Complete information was obtained from 67% of medical oncologists registered in public centres and from 61% of oncologists registered in private centres. The data corresponding to these centres was extrapolated to the 100% of the Register of Medical Oncologists, in cases for which detailed information was not available.

The number of medical oncologists was converted to full time equivalent (FTE), considering part-time contracts, deducting 10% of the correspondent time for women, due to the higher probability of leaves or early departure from the labour market (using estimates from previous studies). [9].

### Estimate of available professionals in the labour market (offer) according to entry and exit

With the aim of estimating the number of specialists who will make up the medical oncology workforce in our country with a time horizon to 2035, a predictive model was designed based on specialist replacement according to the entries (number of trained specialists) and exits (professionals who exit the healthcare delivery labour market for different reasons). The model applied made it possible to quantify the number of specialists in Medical Oncology with care delivery tasks, who will make up the Medical Oncology workforce within the next years, and who could be potentially available to meet healthcare delivery needs.

To estimate the number of entries into the labour market, the average of Resident Medical Intern (MIR)-resident doctor positions published in the Spanish Official Bulletin during the last 5 years was considered (112 positions per year), and this number was considered constant [11].

The following aspects were considered to estimate the number of exits from the labour market: (1) the percentage of trained specialists who work in sectors other than care delivery (research, management, pharmaceutical industry, etc.). This percentage was estimated at 4% based on previous studies [9]; (2) the percentage of interns’ re-circulation (Medical Oncologists who have finished their residency and apply for a new speciality, estimated at 10% based on previous studies [9]), (3) the percentage of interns trained in our country who work abroad after finishing their internships (estimated at 2% of the total of Medical Oncology interns [9]) and (4) the percentage of retirements. To estimate the number of retirements within the time frame of the study, the most probable age of retirement considered was 65 years old, although it was considered that 10% of professionals would continue working until the age of 67.

### Estimation of the number of medical oncologists needed related to workloads

For the estimation of the number of specialists required to fulfil the present and future workloads, the model used was taken from the *First White Book of Medical Oncology in Spain* [12]. The data on the total activity performed by Medical Oncology Departments have been updated to 2013 through information generated by SEOM [13]. In the case of genetic counselling consultation activity, estimated based on data on activity in reference centres, as indicated in Table 1. The optimal time dedicated to each one of the main activities has been updated considering the increase in complexity of care delivery and based on the available literature on the subject [14, 15]. Specifically, regarding time dedication included in the First White Book of Medical Oncology in Spain, the time dedicated to the first visit has been increased from 45 to 60 min, and the second visit has been considered in a differential way from the rest of subsequent consultations, estimating optimal dedication at 90 min. We also considered new activities (not included in previous studies), which represent a growing proportion of total medical oncologist’s dedication time, such as tele-consultations (estimated dedication of 7.5 min/consultation) and the genetic counselling (90 min estimated for the first visit and 30 min for subsequent visits). The optimal dedication time of the medical oncologist for the first genetic counselling visit was estimated considering the consultation time and the requirement of later dedication for the computerization or analysis of genealogical tree, etc. The optimal times estimated by type of activity and the sources used for each case are shown in Table 1.

**Table 1:** Activity by Spanish Medical Oncology Departments in 2013, optimal dedication times and total times by type of activity

	Ambulatory care delivery					During hospitalization		On call	Tum. commit.		Genetic counselling	
	First	Sec.	Subseq.	Telec.	DH	Stays	Cons ser.				First	Subseq
Activity (nº)	127,034^a^	127,034^a^	1,533,740^a^	13,515^a^	1,231,067^a^	565,134^a^	33,149^a^	33^a^		57,942^a^	37,250^b^	56,105^b^
Time (min)^c^	60	90	20	7.5	15	20	30	6.264 h/y		60	90	30
Total time (h)	127,034	190,551	511,247	1689	307,767	188,378	16,575	206,712		57,942	55,870	28,053
Total work-time (h) to meet demand	1,691,818											

All consultation data refers to consultations in 2013 by medical oncology specialists

*First* first consultations

*Sec* second consultations

*Subseq* subsequent consultations

*Telec* tele-consultations or consultations not done in person, by oncologists in 2013. Includes consultations resolved with primary care

*DH* day hospital activity. No. of sessions in day hospital in 2013

*Stays* Total number of hospital days (stays) in conventional hospitalization units in 2013, considering those in Oncology Departments beds as well as in other departments in which patients were attended by oncologists

*Cons ser* consultation services (with other specialists). Number of hospital inter-consultations in 2013

*On call* ongoing attention. Number of on-call positions located in oncology (excluding residents). To estimate the total hours per year required to cover ongoing attention, 130.5 h per week (time not covered by a normal work-day), in 33 on-call positions were considered. The time to cover on-call shifts was not estimated independently, as it has been considered included in the hours estimated to cover the care activities during conventional work hours

*Tum. commit* tumours committee No. of Tumour Committee sessions held in 2013

*h* hours, *min* minutes, *d* days, *y* year

^a^Activity data in Spanish Medical Oncology Departments corresponding to 2013 [13]

^b^Estimate based on data on the number of genetic advice consultations in three specialised oncology centres in Catalonia, extrapolating that data to the population of Spain

^c^Average dedication time of the Medical Oncology speciality [12, 14, 15]

Considering the total activity carried out in Spain in 2013, the optimal dedication times, and the population requiring health services, we have estimated the number of working hours of a medical oncologist needed per 100,000 inhabitants to cover the present demand.

For the estimation of the optimal FTE to cover this demand, the total available time per FTE considered is 37.5 h per week, considering that dedication to healthcare delivery tasks represents, on average, 72% of the total time of the workday. The rest of the time would be distributed between training, research and management activities, depending on profiles.

### Estimation of the number of medical oncologists needed related to the incidence increase

The estimation of the needs of medical oncologists on the basis of optimal ratios of new cases/specialist was done using the incidence projections of the Globocan 2012 Study [4]. In all cases, the needs of specialists were estimated in terms of FTE.

For the estimation of new cases in Medical Oncology Departments, hematologic cancers were excluded, excepting 10% of the new cases of these types of cancer, considered to be attended at oncology offices.

## Identification of future Medical Oncology trends and needs

With the aim of collecting the opinion of the Medical Oncology professionals regarding future trends and needs of the speciality, an online survey was designed and addressed to all the medical oncologists, members of SEOM, with care delivery activity, in the public and/or private sector. The questionnaire collected opinions related to future trends and needs of Medical Oncology, through 24 questions distributed in 2 blocks (Block 1 “Analysis of trends and needs” and Block 2 “The situation in your Department”). The questionnaire used is available for consultation at http://seom.org/adjunt/Cuestionario_Plan_de_Futuro_OM.pdf. 176 medical oncologists participated in the survey, which represents 17.7% of the 993 medical oncologists who were provided with access to the survey. Based on conclusions of the online survey, we worked on the identification of future recommendations through a face-to-face session with the project’s Advisory Committee, a work group formed, for this purpose, by members of SEOM.

## Results

The data from the Register of Medical Oncologists, collected within the framework of the project, show that at present, a total of 1216 medical oncologists (excluding medical residents) work in care delivery centres, 78% in centres of the public National Health System with exclusive dedication; this figure is equivalent to 1141 FTE specialists.

**Fig. 1:**
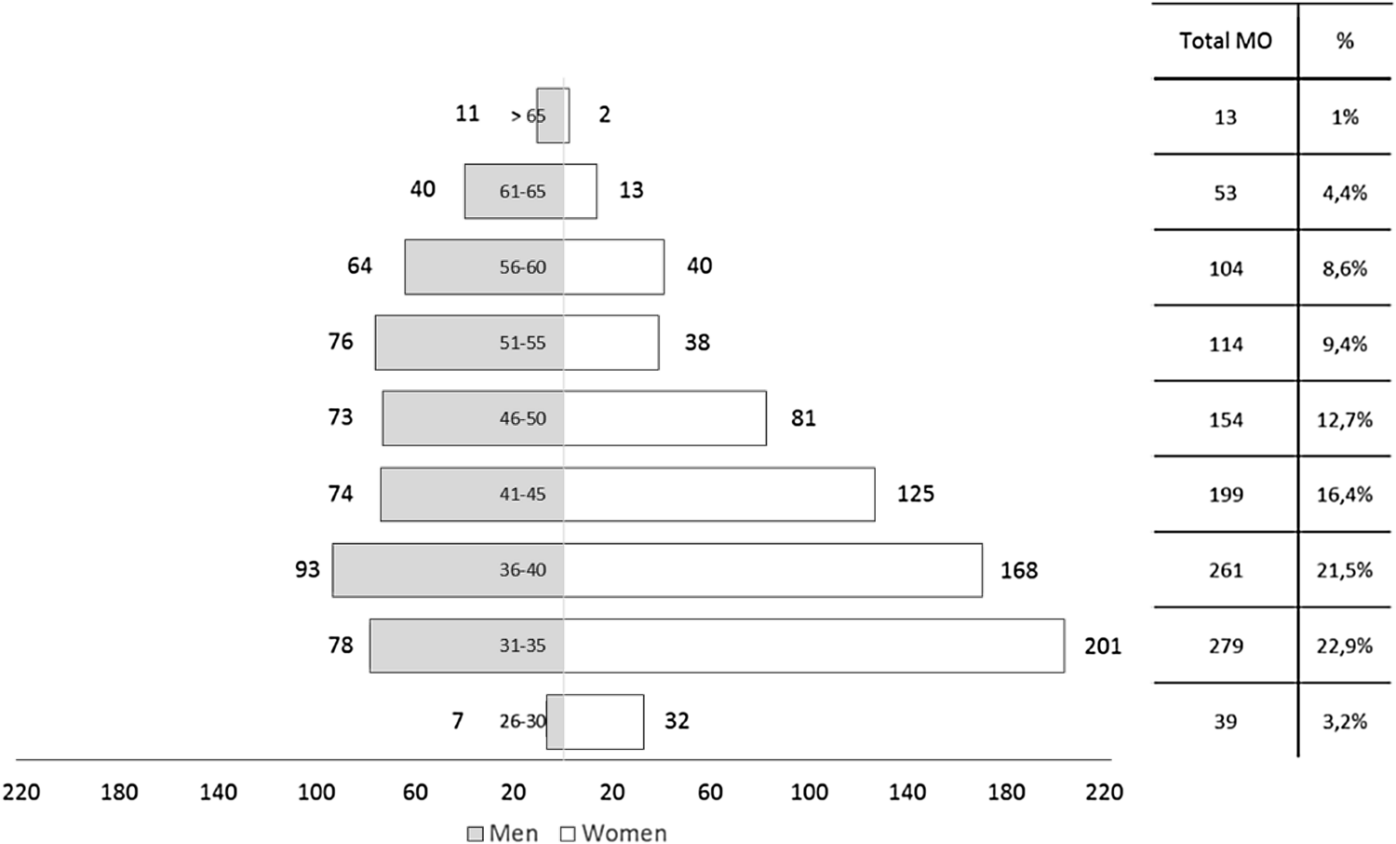
Distribution by age and gender of the Spanish medical oncologists (Register SEOM 2014)

The age pyramid of Spanish medical oncologists (Fig. 1) shows a young speciality, with predominance of women (57.6%). This rate is higher in younger frames (over 70% for oncologists under 35). Only 23.3% of the current medical oncology workforce is over 50 years old. The pyramid of professionals working in the private sector shows a higher rate of men (53.6%) and a higher average age (34% of medical oncologists in the private sector are over 50).

The average number of medical oncologists working in the same department is 6.5. This average is slightly higher in public centres (7 medical oncologists/departments versus 4.6 medical oncologists/departments average in the private sector).

Considering the required dedication to different activities carried out by medical oncologists and the current volume of activity in the departments, a need of 3621 h of dedication of medical oncologists per 100,000 inhabitants (considering Spanish population at 1st January 2013, according to data from the National Institute for Statistics) was estimated. Considering the present number of new cases of cancer (incidence 2013), 822 h would be required per 100 new cases. Considering the average number of annual dedication hours per FTE, 2.79 FTE/100,000 inhabitants or a FTE per 158 new cases would be required to reach these ratios (Table 2).

**Table 2:** Estimation of the needs of Medical Oncology specialists based on workloads

	Total care delivery
Total time (h) for meeting demand	1,691,818
Hours/10^5^ inhab^a^	3621.70
Hours/100 new cases^b^	822.2
No. of FTE needed/10^5^ inhab^c^	2.79
No. of FTE needed/100 new cases^d^	0.634
Optimal rate related to incidence 2015	158 new cases/FTE

^a^To estimate the total hours needed to meet a demand corresponding to 100,000 inhabitants, total hours required to cover the demand for oncology services (1,691,818 h) was divided by the population of Spain as of 1 January 2013 (46.7 million), according to data from the National Institute for Statistics, and multiplying this ratio by 100,000

^b^To estimate the total hours needed to meet a demand corresponding to 100 new cases, incidence projections of the Globocan 2012 Study [4] were used

^c, d^Conversion of total hours into full-time equivalents were obtained considering 37.5 h/week as a FTE. According to data collected and empirical experience from work group, it was estimated that 72% of the workday is dedicated to direct patient care. This accounts for 1296 h/year dedicated to patient care per ETC

**Fig. 2:**
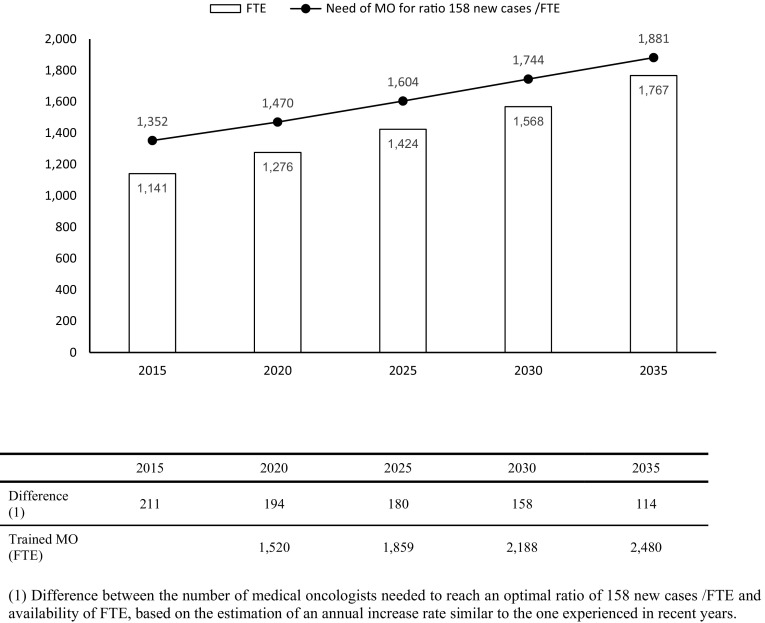
Expected growth of workforce in baseline scenario and estimation of FTE needs to reach ratio of 158 new cases/FTE

To reach the ratio of 158 new cases/FTE and depending on the incidence estimated for 2015, 1352 FTE would be required. Considering that, according to the register, there are 1141 FTE, there would be a deficit of 210 FTE (Fig. 2).

Considering that the medical oncology workforce would maintain an annual average growth of 2.3%, based on previous available studies [9] and the growth rate in SEOM membership, the workforce in 2035 will be of 1767 FTE. Considering the estimates of the number of new cancer cases included in the Globocan study in our country, 1881 medical oncologists FTE would be required to maintain a ratio of 158 new cases/FTE in 2035 (Fig. 2). Considering the estimates of the number of trained specialists in our country, we would be able to meet this demand in 2035.

### Main results of the online survey to medical oncologists on changes, challenges and future needs of Medical Oncology

**Fig. 3:**
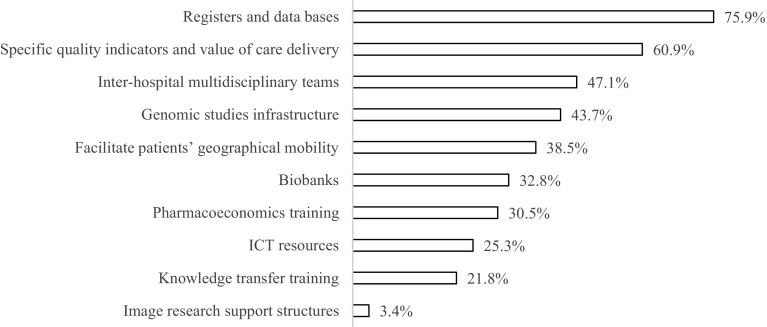
Perception of needs regarding the development of priority research lines and the establishment of innovations based on their value in Medical Oncology. Results from the online survey “present situation, challenges and future needs of MO” carried out in 176 Medical Oncologists. The number of valid surveys was 174. Rate of participants who selected each item among the most relevant 5 is represented. *ICT* information and communication technologies

In the opinion of the professionals surveyed, the aspects that may contribute significantly to improve the quality of care delivery are the development of a national quality assessment system and the development of functional units for specific cancer pathologies. In regard to the relative needs of cancer research and the introduction of innovations on the basis of their value in the area of oncology, it is important to note that 76% of the professionals surveyed request the development of appropriate-for-decision-making registers and databases, and 61% report the need for the development of specific indicators for assessing the quality of results and managing the incorporation of new evidence-based therapeutic approaches (Fig. 3).

In this sense, according to the data collected in this survey, 85.8% of oncologists do not have data demonstrating the impact in health outcomes derived from organizational changes and from the broadening of their service portfolios, and only 10.9% of professionals report having participated in studies on health outcomes. In addition, 67.1% feel that their department does not include an appropriate panel of indicators for the assessment of quality of care delivery.

The main concerns, challenges and needs of the speciality reported by the specialists surveyed have been grouped in 5 broad areas: (1) adapting Medical Oncology workforce to new needs; (2) developing specific care delivery plans adapted to new profiles and their specific needs; (3) ensuring the equity and access to quality healthcare delivery; (4) ensuring earlier diagnosis and (5) advancing towards a more accurate and comprehensive cancer care delivery. A total of 29 recommendations have been identified (Table 3).

**Table 3:** Recommendations included in the Future Plan of Medical Oncology

Areas	Recommendations	
**Area 1** Adapting Medical Oncology workforce to new needs	R1	Adapt the number of MO to the growing needs of cancer care delivery and higher complexity of patients
	R2	Adapt average dedication time per patient, especially in first and second consultations as well as time dedicated to research
	R3	Create a Register of professionals that facilitates ongoing analysis and assessment of specialists’ needs based on demand changes
	R4	Reduce MO’s administrative workload, by strengthening administrative support resources
	R5	Concentrate high complexity care, ensuring optimal resources for its management
	R6	Adapt continuous training and Specialised Healthcare Training programmes to the speciality’s challenges: biomarkers and precision medicine, multidisciplinary work skills
	R7	Strengthen MO contents included in bachelor programmes with a higher involvement of bachelor MO students
	R8	Promote super-specialisation in care delivery teams
**Area 2** Developing specific plans of care delivery adapted to new profiles and their specific needs	R9	Promote the participation of MO in the development of specific plans for long-term survivors
	R10	Promote the participation of MO in the development of specific plans for ongoing care
	R11	Promote the participation of MO in the development of specific plans for care delivery to elderly and patients with co-morbidities
	R12	Improve the coordination MO-Primary Care for the patients’ follow-up
**Area 3** Ensuring the equity and access to quality healthcare delivery	R13	Establish healthcare outcomes assessment and dissemination systems. Creation of a national quality assessment system
	R14	Develop clinical information and treatment plan registers with access for patients
	R15	Advance in the definition of agreed protocols/therapy guidelines and promote their implementation
	R16	Define, establish and measure useful indicators for the assessment of care delivery quality and the impact of innovation incorporation
	R17	Facilitate the access of MO to systematic quality assessment tools through actual outcomes that allow the integration of care delivery, resources management, economic and health outcome data. Outcome assessment in terms of effectiveness
	R18	Promote participation of Oncology Departments in quality accreditation systems
**Area 4** Ensuring earlier diagnosis	R19	Improve coordination MO-Primary Care. Improve resources and training for an early diagnosis
	R20	Ensure the access to early detection strategies with evidence-based usefulness specially in colorectal, lung breast, cervix and prostate cancer
	R21	Promote research in early stages of the disease
**Area 5** Advancing towards a more accurate and comprehensive cancer care delivery	R22	Promote the creation of multidisciplinary teams for all kinds of cancers
	R23	Promote the creation of inter-hospital joint services (care delivery, training and research in collaboration)
	R24	Promote the creation of centres based on the philosophy of *Comprehensive Cancer Centre,* which integrate multidisciplinary care delivery, training and research in cancer
	R25	Increase the use of ICT for promoting inter/multidisciplinary work
	R26	Promote research in biomarkers, immunotherapy and combined therapies
	R27	Promote network research for increasing its scope
	R28	Facilitate the inclusion of patients in clinical trials through the use of ICT
	R29	Promote the creation of patient registers and ensure optimal exploitation of electronic medical history data

*MO* medical oncology or medical oncologists, *ICT* information and communication technologies

#### Area 1: adapting the medical oncology workforce to new needs

The optimal ratios for resources planning must be based on workload. This load must be estimated considering the appropriate dedication time to the different types of activities carried out by medical oncologists (especially to second consultations and consultations with patients included in clinical trials). This adjustment in dedication time must take into account changes in the patient profiles, the increase in prevalence and complexity, and the development of new services and activities, such as the interpretation of diagnostic tests. The development of a Register of Professionals that allows ongoing analysis and forecasting of the needs of specialists is another recommendation in this area.

#### Area 2: developing specific care delivery plans adapted to new profiles and their specific needs

The main recommendations in this area are to define and organise the participation of medical oncologists in the application of the specific plans for long-term survivors, palliative care and the development of specific plans for special populations (elderly and patients with co-morbidities). Specifically, care delivery to a growing number of long-term cancer survivors is considered one of the main challenges for the speciality. One of the improvement areas reported is the development of more effective coordination strategies with Primary Care, including a coordinated treatment plan that meets the needs of this group of patients, to allow more effective follow up and avoid duplication of tests.

#### Area 3: ensuring equality and access to quality healthcare delivery

Equal access to quality health services must be grounded on an adequate measurement of health outcomes obtained by the system, with quality indicators that allow the comparison between services and systems to determine which actions and structure, organizational or treatment changes are contributing the most to the system. Indeed, the creation of a national system of care delivery quality assessment would be desirable. This would allow more coordinated work, as well as the generalization of systems for the assessment and dissemination of health outcomes.

The contribution of Medical Oncologists to the generation of quality Registers of Patients and Clinical Information, as well as the access to tools for the systematic review of their activity, are considered to be essential for the development of studies that ensure the establishment of more effective strategies. It is also necessary to advance in the definition of accepted protocols and establish the resources required to facilitate their implementation.

#### Area 4: ensuring early diagnosis

The main recommendations in this area include ensuring the access by population to appropriate early diagnosis strategies, especially in breast, lung and colo-rectal cancer, the establishment of tools for the early detection, and referral from Primary Care and the promotion of research on early stages of the disease.

#### Area 5: advancing towards more accurate and comprehensive cancer care delivery

Promotion of research on biomarkers, immunotherapy and combined therapies, and boosting the creation of centres based on the philosophy of *Comprehensive Cancer Centre,* integrating cancer multi-disciplinary care delivery, training and research, are considered essential lines of work to advance in a more and more accurate and comprehensive approach of cancer.

Finally, universal access must be granted, within appropriate times, to the determination of biomarkers of quality and clinical evidence-based usefulness. We, therefore, recommend the creation of a national biomarker platform associated with a specific register.

## Discussion

### Register and future needs of medical oncologists

SEOM decided to promote the elaboration of the Future Plan for Medical Oncology as a means of establishing the speciality’s strategic lines of development in the coming years and address future potential workforce shortages, with the participation and opinion of a large number of the Society’s members.

Both the American Society of Clinical Oncology (ASCO) and the European Society for Medical Oncology (ESMO) have approached this subject through similar studies aimed to identify differences between the actual needs of oncologists with regard to the increase of the Departments’ needs and to the previsions on the number of available professionals, in the basis of the expected workforce growth. According to a study published by ASCO in 2015, it was estimated that demand for medical oncology services would increase by 42% in the USA by 2025. Nevertheless, the expected growth of the oncology and haematology workforce is estimated at 28% for the same period, which means a foreseeable deficit of 1487 FTE oncology/haematology specialists in 2015 [16]. Different studies conducted in the last few years in Australia, Japan, Latin America, France and Belgium, also report an expected shortfall of specialists in the area of oncology within the next years [10]. According to this study published in 2014, the ratio of cancer cases per FTE in 2008 in European countries ranged from 113 in Hungry and 1067 in the United Kingdom, and these ratios are expected to decrease in the coming years in most of the countries. The study reported, however, as its main limitation, the lack of information on professionals in several countries, including Spain [10].

Studies from the University of Las Palmas de Gran Canaria (Spain) on the supply and demand for medical oncologists in Spain have been the main source of information on resource planning for different specialities [9]. Several scientific associations have promoted studies on the needs of different specialists [17, 18], for instance, the Spanish Association of Cardiology reports in its last study the existence of an imbalance of around 14% between the number of active cardiologists and the number needed [17].

Due to the lack of updated data, and in expectation of the results from the unified Register of Professionals, we decided to prepare our own register, with the aim of determining the current situation of Medical Oncology Departments related to the medical oncology workforce in Spain. It was also considered necessary to estimate the optimal ratios based on the increase and complexity of the current demand, and to compare the data obtained with published optimum ratios.

According to the data from published international standards, the optimal range of new cases/FTE is estimated to be between 150 and 180 [19]. In our study, the data from the Register of Medical Oncologists show a deficit of 211 FTE to achieve optimal ratios, estimated at 158 new cases/FTE, which means an imbalance of 18%. Our study also shows a lower average size of the Departments’ workforce, which is around 6.5 medical oncologists/Department compared to data from other countries such as USA where the average is 15 specialists/Department [16]. Our study also reports the concern regarding the dispersion and breakup of Departments experienced in recent years. This fact has been identified as an element that hinders the concentration of a sufficient number of cases to ensure equality in healthcare delivery quality.

The ageing of the population will foreseeably result in an increase in demand for oncology services. In 2012, 60% of the new cases of cancer in Spain corresponded to patients 65 years of age or older. This rate is expected to increase to 67% in 2035 [4]. Despite the expected decrease in the Spanish population within the next 10 years, the ageing trend will be maintained, and the number of people aged 65 or over is estimated at more than 12.5 million by 2025, an increase of 56% from 2012 [20]. Due to the difficulty of estimating activity figures corresponding specifically to the older population, we opted to use ratios in comparison with the general population, allowing that this aspect may be generating an underestimation of the demand in the coming years. Also, the incidence projections, used to estimate future needs, include the foreseeable ageing of the population in Spain in the year to come.

To attain the optimal number of 1881 FTE by 2035, the medical oncology workforce should present an annual average growth of 2.5%. According to a study in 12 European countries, the average workforce growth was 5.3% (balancing in a range between 1.8 and 8.7%) [10], so that the estimated growth rates for Spain would be within this range. The difference between the number of professionals trained in Medical Oncology and the expected retirements, would make it possible to cover this workforce growth, if the current number of internship positions remain constant, as it is estimated that 2480 medical oncologists would be available by 2035 (Fig. 2).

The main limitation identified in the study is the possible margin of error derived from the estimates of the register due to the lack of complete data from all regions, and the forecasts regarding the estimation of the number of future cancer cases. Although the estimated needs based on the forecast of the number of cases is considered a valid model, the estimation of prevalence projections may be a more precise model for estimating the actual workload.

In regard to the updating of workloads, the lack of information on the rate of consultations with patients included in clinical trials, or the time dedicated to home care, is also a limitation. Despite the estimated maintenance of 72% of time dedicated to care delivery, based on the need for increased involvement by medical oncologists in research, training and management, we recommend that this rate be revised downward to allow time to be allocated to these tasks.

In the coming years, there may be an increase in absences due to common illness, and the workforce needs may increase due to burn-out syndrome. These two factors would offset the expected reduction in maternity leave considered in the model. In this sense, a report prepared by ASCO in 2012 indicated that 44.7% of oncologists/haematologists in the US said that they suffered burn-out, especially in profiles with greater dedication to care activity. Close to 27% of oncologists said that they would like to reduce their work hours over the next 12 months, 34% would consider leaving their current job, and 28% planned to retire before the age of 65.

In a scenario of regulatory changes related to requirements for ordinary, partial, and early retirement, their different application at the regional healthcare services level and the diverse models for the hiring of medical professionals applied in recent years in Spain, an average retirement age of 65 was adopted. Even admitting that this estimate could cause a potential underestimation of the number of professionals in active service in the coming years, the possible impact of other factors, such as burn-out or possible budgetary limitations, which could lead to an increase in early retirement, must be taken into consideration.

### Concerns, challenges and needs of Medical Oncology

The low participation in studies on health outcomes was one of the most remarkable conclusion of the survey (only 10.9% of those surveyed reported that they had participated in studies of this kind, despite the acknowledgement of their importance in the planning and incorporation of innovations to the National Health System). Although the available information is limited, the main programmes of comprehensive oncology applied in different countries acknowledge the need to generate evidence that proves its increased effectiveness in comparison with traditional models of cancer approaches. It is important to carry out a continuous assessment of key indicators related to control of symptoms, adherence to treatment protocols, quality of life, individual outcomes, prevention, rehabilitation, economic impact in services, and considerations regarding the impact on the health system as a whole [21].

## Conclusions

The estimation of the current workload of medical oncologists based on the current service demand and the required dedication to the main activities made it possible to estimate that an optimal ratio of 158 new cases per FTE should be reached. At present 1352 FTE would be required to reach this optimal ratio, versus the 1141 FTE available, which means a deficit of 211 FTE. 1881 FTE would be required to reach the optimal ratio in 2035, which would involve an average annual workforce growth of 2.5%.

Based on the needs and challenges identified by medical oncologists, SEOM would like to highlight the need to progress towards the establishment of actions that: (1) move forward in regard to improvement of healthcare delivery, equal access to care delivery, and increased effectiveness by promoting health outcomes assessment, (2) build more accurate quality indicators, useful for decision-making in the area of medical oncology and (3) ensure that the size of the medical oncology workforce will be appropriate in size to address future demands.
